# Upregulated m7G methyltransferase METTL1 is a potential biomarker and tumor promoter in skin cutaneous melanoma

**DOI:** 10.3389/fimmu.2025.1575219

**Published:** 2025-05-15

**Authors:** Luling Xia, Ping Yin

**Affiliations:** ^1^ Department of Pulmonary and Critical Care Medicine, The Third Xiangya Hospital, Central South University, Changsha, China; ^2^ Department of Blood Transfusion, The Third Xiangya Hospital, Central South University, Changsha, China

**Keywords:** METTL1, SKCM, biomarker, immunotherapy, prognosis

## Abstract

The m7G methyltransferase METTL1 has been implicated in the occurrence and progression of several cancers. However, its clinical significance in cutaneous melanoma (SKCM) remains poorly understood. To address this gap, we conducted comprehensive data mining using publicly available datasets and two single-cell datasets. Additionally, we employed CCK8 assays, clone formation assays, and cell migration and invasion experiments to validate our findings from the data mining. Our results revealed that METTL1 is significantly upregulated in SKCM and is associated with a stem cell-like phenotype. Patients with high METTL1 expression exhibited worse prognosis. Furthermore, we identified that the high expression of METTL1 in SKCM is driven by copy number amplification and regulated by the transcription factor MYC. *In vitro* cellular studies confirmed that METTL1 knockdown significantly inhibited SKCM cell proliferation, clone formation, migration, and invasion. Notably, we observed a strong negative correlation between METTL1 expression and CD8+ T-cell infiltration in SKCM tissues. Moreover, our analysis revealed a significant negative correlation between METTL1 expression levels and the response to immunotherapy in SKCM patients, suggesting that METTL1 may serve as a potential biomarker for predicting immunotherapy response in SKCM. In summary, this study enhances our understanding of the role of m7G RNA modification in tumor progression and highlights METTL1 as a novel therapeutic target and biomarker for SKCM immunotherapy.

## Introduction

METTL1 (Methyltransferase-like 1), located on chromosome 12q13, is a protein-coding gene that has methyltransferase activity in cells (1). Normally, METTL1 is expressed in kidney, thyroid, skin, and 25 other tissues (2). METTL1 functions primarily in the nucleus and is involved in epigenetic modification of RNA. m7G tRNA modification mediated by METTL1/WDR4 is essential for common RNA translation, regulation of cellular self-renewal, and differentiation (3). m7G is one of the most common RNA epitope modifications, often located in the 5’ cap and internal positions of eukaryotic mRNAs, or within rRNAs and tRNAs in all species (4). m7G This modification helps to maintain the stability, proper folding, and functioning of tRNAs, and affects the process of protein synthesis (5). In addition, METTL1 activity may also have some effect on other RNA molecules, such as rRNAs and microRNAs (6). However, the function and regulatory mechanisms of METTL1 still require further studies to fully understand its role in cell biology and disease development.

Previous studies have shown that aberrant expression of METTL1 is closely associated with tumor development and patient survival. For example, METTL1 is associated with poor prognosis in bladder cancer, and it regulates the translation of EGFR/EFEMP1 by modifying certain tRNAs to inhibit the proliferation, migration and invasion of bladder cancer cells (7). In prostate cancer, METTL1 promotes tumorigenesis through tRNA-derived fragment biogenesis, and inhibition of METTL1 activity leads to favorable changes within the tumor, such as an increase in anti-tumor cytokines and infiltration of cytotoxic immune cells, including M1 macrophages and CD8+ T cells (8). METTL1 deficiency leads to reduced abundance and cell cycle alterations of m7G-modified tRNAs, particularly Arg-TCT-4-1, and inhibits oncogenicity (9). In addition, METTL1-mediated tRNA m7G modifications promote the translation of mTOR pathway components, thereby facilitating mTOR activation and progression of esophageal squamous cell carcinoma (10). These studies imply an important role for METTL1 in cancer development. However, its specific role and regulatory mechanism in the progression of cutaneous melanoma have not been clearly reported.

This study is based on public data mining in The Cancer Genome Atlas (TCGA) and Gene Expression Omnibus (GEO) databases, and systematically analyzes the expression, regulation, and clinical value of METTL1 in SKCM, providing reference for prognosis judging and personalized therapy of SKCM.

## Materials and methods

### Databases and data mining and analysis

The databases used in this study include, Kaplan-Meier Plotter, GEPIA2 database (http://gepia2.cancer-pku.cn/#index), BEST database, The Human Protein Atlas database, ASSISTANT for Clinical Bioinformatics tool, cBioportal database, TISCH database, UCSC genomics, and two single-cell datasets (GSE72056 and GSE174401). The Kaplan-Meier Plotter, GEPIA2 database, BEST database, Human Protein Atlas database, ASSISTANT for Clinical Bioinformatics tool, and the cBioportal database were used to analyze the correlation between METTL1 expression and clinicopathological characteristics of SKCM patients. Two single-cell sequencing datasets, GSE72056 and GSE174401, were used to analyze the relationship between METTL1 and tumor cell trajectory by the algorithm of monocle, and the differentiation potential of cells by the CytoTRACE algorithm. ASSISTANT for Clinical Bioinformatics tool analyzed the correlation between METTL1 and stem cell-like phenotype of SKCM and with immune cells. cBioportal database analyzed the correlation between METTL1 copy number amplification and clinical characteristics. The spatial transcriptome data of formalin fixation and paraffin embedding samples on 10x Genomics (https://www.10xgenomics.com/cn/datasets/human-melanoma-if-stained-ffpe-2-standard) were used to analyze the colocalization of METTL1 with SKCM stem cell markers SOX10 and ABCB5. All analyses were conducted by simply selecting the disease type (SKCM in this study) in the database, followed by filtering and extracting relevant data according to the default parameters of the database.

### Cell culture and treatment

The cell lines A875 and A375 were gifts from the Department of Dermatology, Xiangya Hospital, Central South University (Changsha, China), and were cultured in 1640 complete medium with 10% Fetal Bovine Serum (cat no. A2720801, Gibco, USA) at 37°C, 5% CO2 incubator.

### siRNA transfection

To knock down METTL1, A875 and A375 cells were transfected 20 nM siRNA (siRNA sequences: METTL1 #1: GATGACCCAAAGGATAAGAAA; METTL1 #2: GGATGTGCACTCATTTCGA or empty plasmids (control) that mixed with Lip3000 for 48 hours. The transfected cells were used for further analysis.

### Quantitative real time PCR

The transfected cells were collected and added TRIzol reagent to extract the total RNA. Ultra Micro Nucleic Acid Analyzer (NANODrop2000, Shimadzu, Japan) was used to determine the concentration and purity of RNA to meet the requirement of A260/A280 in the range of 1.8-2.0. The extracted RNA was used as the template for reverse transcription to synthesize the cDNA. The real-time fluorescent quantitative PCR (7300 plus, ABI, USA) was used to perform real-time quantitative amplification. The reaction conditions were as follows: 95°C for 30 s, 95°C for 5 s, 55°C for 30 s, 72°C for 30 s, 40 cycles; 95°C for 15 s, 60°C for 1 min, 95°C for 15 s. The specificity of the primers was analyzed according to the melting curves, and the relative expression of the target genes was calculated by using the 2^-ΔΔCt^ method with GAPDH as the internal reference. The primer sequences were as follows:

METTL1-F: AAAGGGGACATGAAAGGGCAA,

METTL1-R: CACCAGACAGACCAAGATGGAA;

SOX10-F: GAGGCTGCTGAACGAAAGTG,

SOX10-R: GCTCTTGTAGTGGGCCTGGA;

CD4-F: GGGATACAGTGGAACTGACCTG,

CD4-R: CAGAGTTGGCAGTCAATCCGAA;

CD8A-F: ATGGCCTTACCAGTGACCG,

CD8A-R: AGGTTCCAGGTCCGATCCAG.

GAPDH-F: AATGGGCAGCCGTTAGGAAA,

GAPDH -R: GCGCCCAATACGACCAAATC.

### Clone formation

A875 and A375 cells were inoculated in 35 mm cell culture dishes (300 cells/dish) and incubated in an incubator for 2 weeks, with medium changes every 2 day. The supernatant was washed 3 times with PBS and fixed with methanol for 15 min, incubated with 2 mL of crystal violet staining solution for 30 min, washed 3 times with PBS, and air dried. When the diameter of cell clone was >0.75 mm, it was recognized as positive and manually counted.

### RNA-seq data analysis

The METTL1 siRNA transfected A875 cells and control cells were collected and used for sequencing in BGISEQ-500 platform (Beijing Genomics institution, Shenzhen, China). mRNA was enriched using oligo(dT) magnetic beads and denatured to open its secondary structure. The mRNA was fragmented and used to synthesize double-strand cDNA to amplify. After denaturing the PCR product into single-stranded, the cyclization reaction system was performed to obtain the single-stranded cyclic product, and digest the linear DNA molecules that have not been cyclized. The single-stranded cyclic DNA molecules were used for rolling circle replication to form a DNA nanoball (DNB) containing multiple copies, which were sequenced by co-probe anchored polymerization (cPAS). The raw data obtained from sequencing was filtered using SOAPnuke (v1.5.6) and clean data was compared to the reference gene set using Bowtie2 (v2.3.4.3). Gene expression was quantified using RSEM (v1.3.1) software and differential gene detection was performed using DESeq2 (v1.4.5), and the heatmap was displayed. Gene function analysis was based on GO.

### CCK8

METTL1 siRNA and negative control were transfected into A875 and A375 cells respectively for 24 h. After transfection, the cells were seeded into 96-well plates at 5×10^3^ cells per well, and 75 uM photosan was added into each well after the corresponding time points (0, 1, 2, 3, 4 day), and the cells were subjected to a 630 nm laser treatment for 4 h. 10 μl CCK8 was added into each well after 24 h. The OD value of CCK8 was determined using a 450 nm wavelength and the growth curve was plotted.

### Cell migration and invasion

The transfected cells were removed from the complete culture medium, replaced with serum-free medium for 24 h. The cells were digested and collected into centrifuge tubes, washed twice with PBS, and the cells were suspended in basal medium and precipitated. 5×10^4^ cells were added to the upper chambers, and then supplemented with serum-free medium, and fresh complete medium was added to the lower chambers of the chambers. The chambers with matrix gel were used to detect cell invasion, and the chambers without matrix gel were used to detect cell migration. The cells were put into the incubator for 48 h. The liquid in the chambers was wiped out by cotton swabs and put into paraformaldehyde solution for 30 min; the chambers were clamped out, the liquid in the chambers was poured out, and the chambers were stained with crystal violet staining solution for 20 min; the chambers were put into ultrapure water for rinsing, and the liquid in the chambers was discarded, and the liquid was dried out upside down; the chambers were photographed under a microscope for cell counting and analyzing.

### Cell cycle detection by flow cytometry

A875 cells were transfected with METTL1 siRNA and negative control respectively, and then cultured for 72 h after transfection, and then the cells were collected and counted. 1×10^5^ cells were obtained and washed by pre-cooled PBS, and then were resuspended by adding 70% ethanol solution to the cell precipitates, fixed for 30 min, added with PI staining solution, and incubated for 30 min away from light, and then detected by flow cytometry.

### Statistical analysis

All experiments were repeated three times, and the data are expressed as the mean ± standard deviation. Statistical analysis was performed using GraphPad Prism 8 software. A t test was used for comparisons between two groups. For multiple comparisons involving more than two groups, Tukey’s *post hoc* test and one-way analysis of variance (ANOVA) were used. *P* < 0.05 was regarded as statistically significant difference.

## Results

### Upregulated METTL1 is an independent prognostic indicator of SKCM

**Figure 1:**
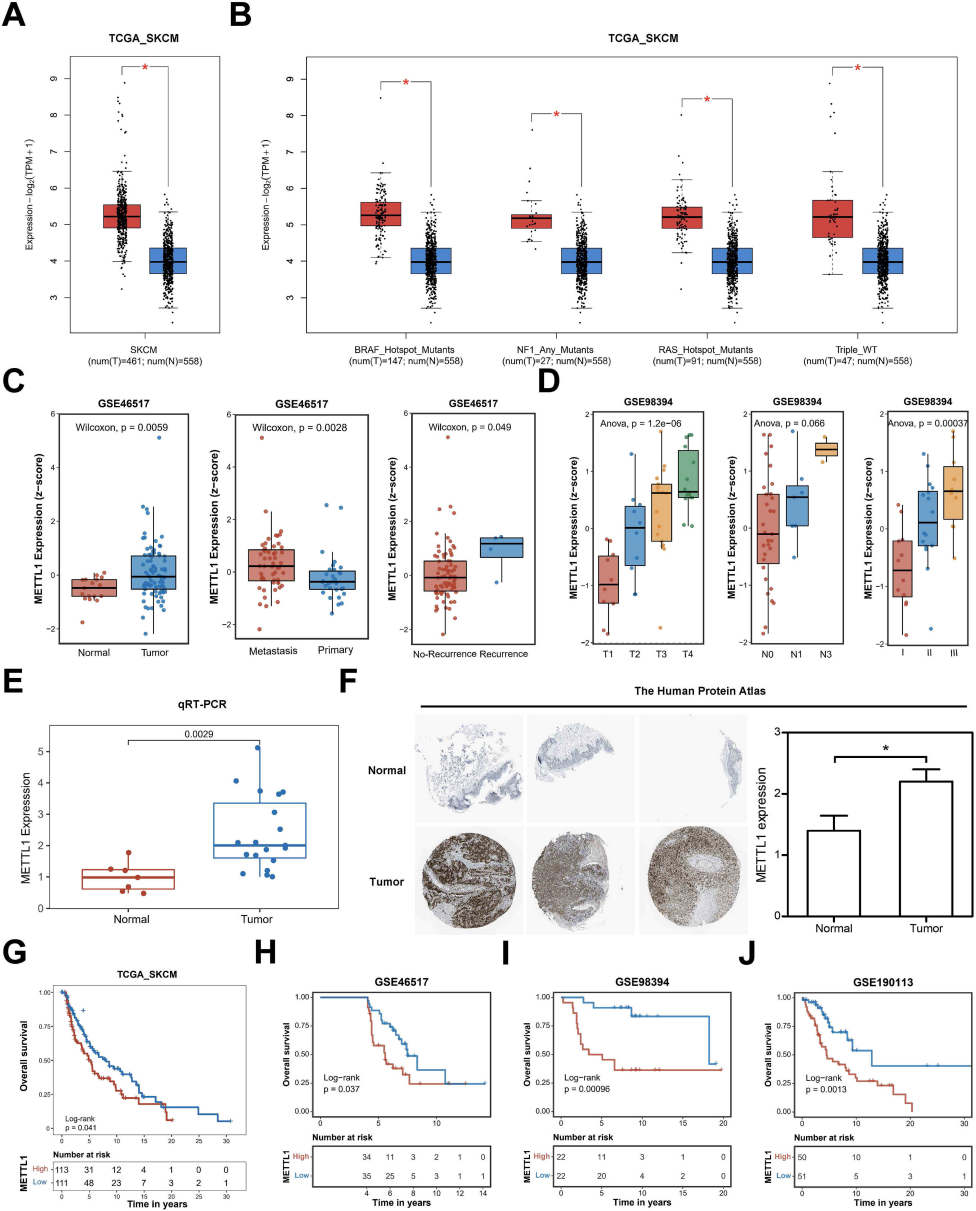
The association of high expression of METTL1 with clinicopathologic features of the SKCM patients. **(A)**, the GEPIA2 online database analyzed the expression of METTL1 in the TCGA SKCM cohort. **(B)** Differential analysis of SKCM genomic variants according to their type into four subclasses, BRAF mutated, NF1 mutated, RAS mutated, and wild-type without any of the three mutations. **(C)** METTL1 expression was analyzed in GSE46517, another independent SKCM cohort dataset, including metastasis, recurrence tumor, primary, and no-recurrence tumor. **(D)** METTL1 expression was analyzed in another independent SKCM dataset GSE98394 with T grade, N grade, and clinical stage. **(E)** the mRNA level of METTL1 was validated in SKCM tissues (N=19) and normal tissues (N=7) by qRT-PCR. **(F)** Immunohistochemical staining of METTL1 in melanoma and normal tissues (N=5) from The Human Protein Atlas (METTL1 antibody, 1:400, cat no. HPA020914, Sigma-Aldrich) and quantification. **(G)** Survival analysis showed the overall survival of patients with high or low METTL1 expression in the TCGA_SKCM cohort. **(H–J)** Survival analysis showed the overall survival of patients with high or low METTL1 expression in the three other SKCM cohorts, GSE46517, GSE98394, GSE190113. *P < 0.05.

Using the GEPIA2 online database (11), we first analyzed the expression of METTL1 in the TCGA SKCM cohort and found that METTL1 was significantly upregulated in SKCM (**Figure 1A**). Differential analysis of SKCM genomic variants according to their type into four subclasses, BRAF mutated, NF1 mutated, RAS mutated, and wild-type without any of the three mutations, revealed that METTL1 was significantly upregulated in all four types of SKCM samples (**Figure 1B**). However, there was no significant difference in METTL1 expression in these mutant types of SKCM (**Supplementary Figure S1A**). METTL1 expression was also similarly markedly upregulated metastasis and recurrence tumor compared with primary and no-recurrence tumor in GSE46517, another independent SKCM cohort dataset (**Figure 1C**). In another independent SKCM dataset GSE98394, the expression of METTL1 increased with the increase of T grade, N grade, and clinical stage (**Figure 1D**). Subsequently, we also validated that the mRNA level of METTL1 was higher in SKCM tissues (N=19) compared with normal tissues (N=7) by qRT-PCR (**Figure 1E**), and similar results were observed in immunohistochemical staining from The Human Protein Atlas similarly (**Figure 1F**).

Furthermore, we analyzed the correlation between METTL1 and the clinicopathological features of SKCM patients. Survival analysis showed that patients with high METTL1 expression in the TCGA_SKCM cohort had shorter overall survival (**Figure 1G**), and consistent results were also obtained in the data from the three other SKCM cohorts, GSE46517, GSE98394, GSE190113, and GSE19234 (**Figures 1H–J**, **Supplementary Figure S1B**). In the following, we found that patients with high METTL1 expression in the GSE133713 dataset had lower recurrence-free survival (**Supplementary Figure S1C**). According to these survival curve results, METTL1 may function as an independent prognostic indicator for SKCM patients. This evidence implies that METTL1 may play a critical role in the development and progression of SKCM.

### METTL1 is expressed in stem-like SKCM cells at early stage of differentiation

**Figure 2:**
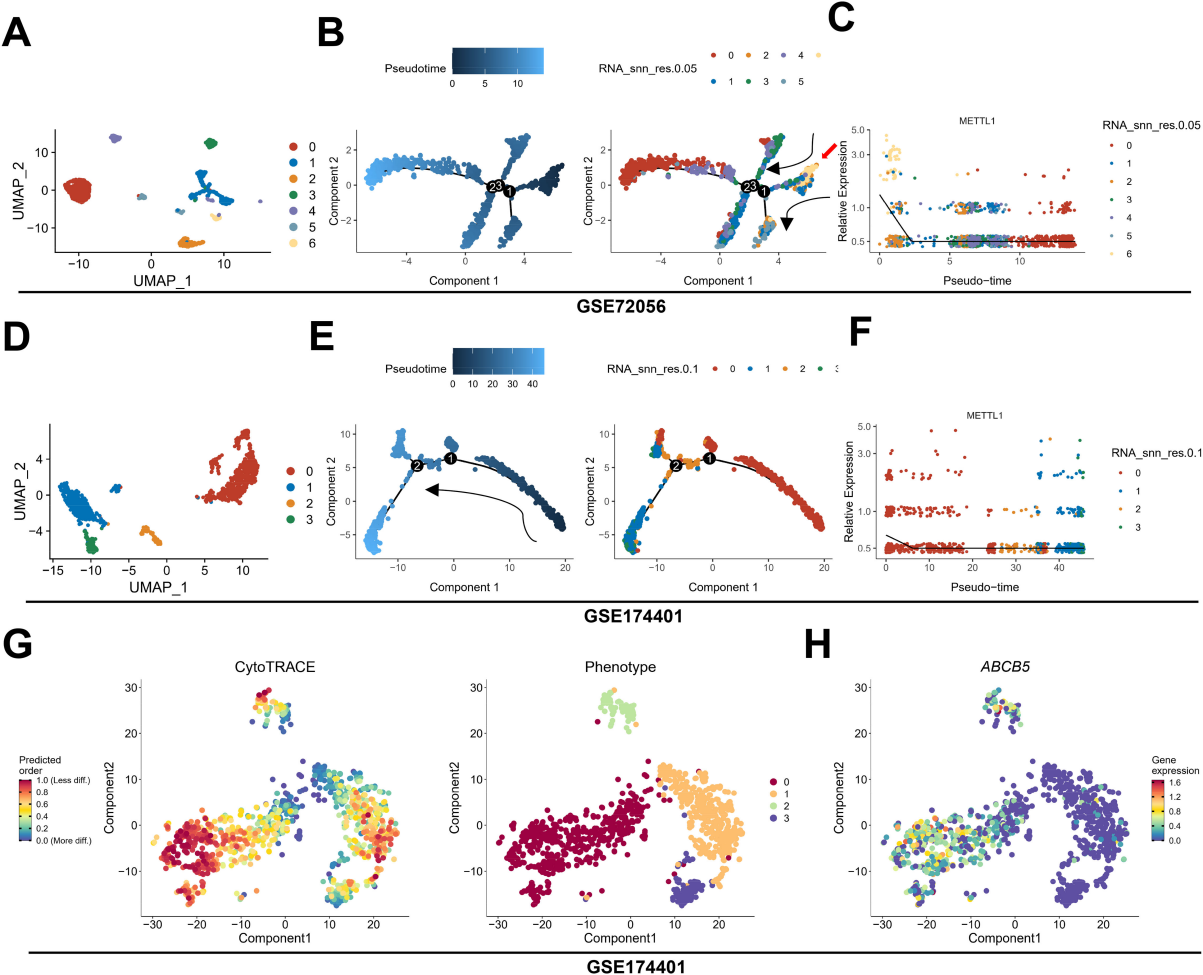
The relationship between METTL1 and tumor cell trajectories. **(A–F)** using monocle algorithm, the relationship between METTL1 and tumor cell trajectories was analyzed in two sets of single-cell sequencing data, GSE72056 **(A–C)** and GSE174401 **(D–F)**. **(G)** the CytoTRACE algorithm was used to analyze the differentiation potential of the cells. **(H)** ABCB5, an SKCM stem cell marker was highly expressed in Cluster 0.

**Figure 3:**
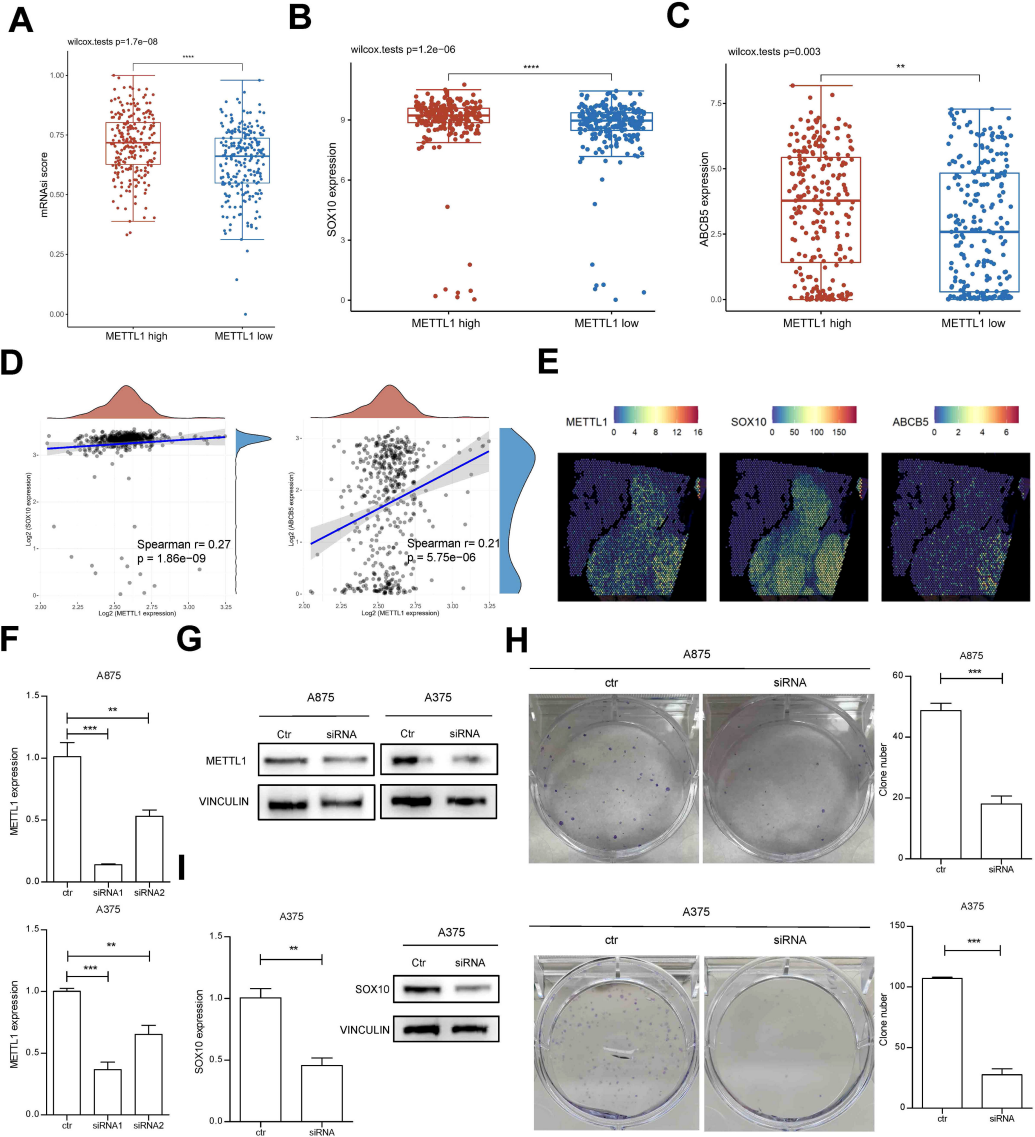
the relationship between METTL1 expression and cell stemness. **(A)** The ASSISTANT for Clinical Bioinformatics tool was used to analyze the relationship between METTL1 expression and cell stemness mRNAsi score. **(B, C)** The SKCM stem cell marker genes SOX10 and ABCB5 were highly expressed in high METTL1 group. **(D)** METTL1 expression was significantly and positively correlated with SOX10 and ABCB5. **(E)** spatial transcriptome data was used to analyze the co-localization of METTL1 and stem cell marker genes SOX10 and ABCB5. **(F, G)** A875 and A375 SKCM cells were transfected with METTL1 siRNA and qPCR and western blot were performed to detect METTL1 expression. **(H)** the clone formation assay was performed to evaluate the functions of METTL1 on clone formation ability. **(I)** qPCR and western blot were performed to detect SOX10 expression after METTL1 knockdown. **P < 0.01, ***P < 0.001, ns, no significant.

To further determine the spatiotemporal expression pattern of METTL1, we analyzed the relationship between METTL1 and the developmental chronology of tumor cells using monocle algorithm with two sets of single-cell sequencing data, GSE72056 and GSE174401. The analysis revealed that METTL1 was highly expressed in cluster6 in GSE72056, cluster 6 was located at the beginning of the trajectories (**Figures 2A–C**), and cluster 6 was significantly enriched for stem cell-associated MYC, Hedgehog, and WNT-β-catenin signaling pathways (**Supplementary Figures S2A, B**). Similarly, METTL1 was highly expressed in cluster 0 in GSE174401, which was also located at the beginning of the trajectories (**Figures 2D–F**). In addition, the differentiation potential of the cells was analyzed using the CytoTRACE algorithm, and it was found that cluster 0 had the highest differentiation potential and highly expressed ABCB5, an SKCM stem cell marker (**Figures 2G, H**). Analysis of the relationship between METTL1 expression and cell stemness scores using the ASSISTANT for Clinical Bioinformatics tool found that the mRNAsi score was higher in the METTL1 high expression group (**Figure 3A**), and the SKCM stem cell marker genes SOX10 and ABCB5 were highly expressed (**Figures 3B, C**), and METTL1 expression was significantly and positively correlated with these two marker genes (**Figure 3D**). In addition, spatial transcriptome data analysis revealed that METTL1 expression co-localized with stem cell marker genes SOX10 and ABCB5 (**Figure 3E**).

We further confirmed the function of METTL1 by *in vitro* cellular experiments. Knockdown of METTL1 in SKCM cells A875 and A375 by siRNA transfection and siRNA1 was selected for further analysis (**Figure 3F**). After METTL1 siRNA transfection, the protein expression of METTL1 was significantly knocked down (**Figure 3G**). Downregulation of METTL1 significantly inhibited the clone formation ability of SKCM cells (**Figure 3H**) and decreased the expression of stem cell marker gene SOX10 (**Figure 3I**). These results suggest that the function of METTL1 is closely related with stem cell-like SKCM cells.

### Knockdown of METTL1 inhibits migration and invasion of SKCM cells

**Figure 4:**
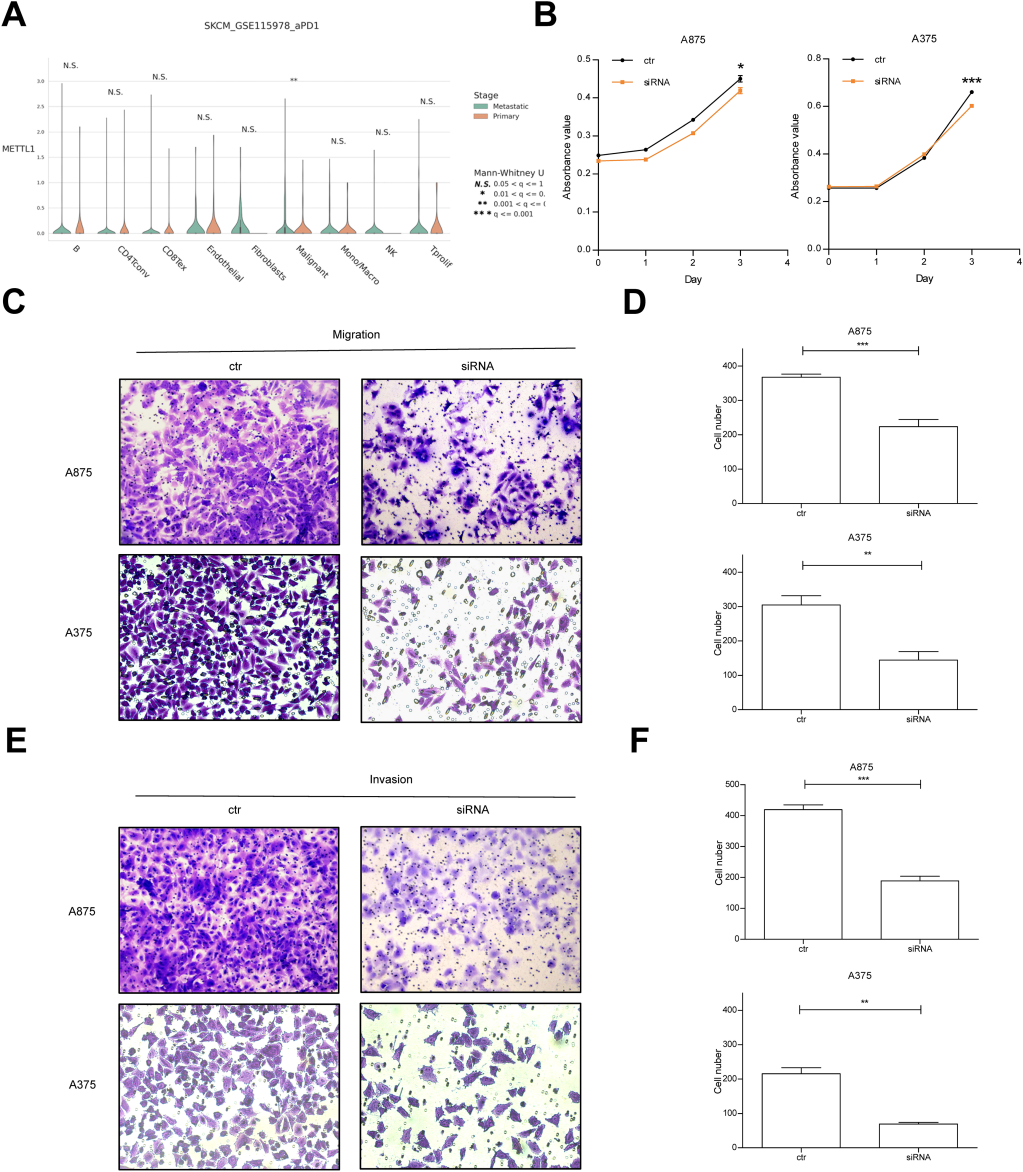
Knockdown of METTL1 inhibits migration and invasion of SKCM cells. **(A)** TISCH database was used to analyze the METTL1 expression in various cells of primary and metastatic tumor. **(B)** CCK8 assay was performed to evaluate the cell viability of A875 and A375 cells after METTL1 knockdown. **(C, D)** Transwell assay was performed to evaluate the cell migration of A875 and A375 cells after METTL1 knockdown. **(E, F)** Transwell assay was performed to evaluate the cell invasion of A875 and A375 cells after METTL1 knockdown. *P < 0.05, **P < 0.01, ***P < 0.001.

TISCH database analysis showed that METTL1 expression was higher in metastatic malignant tumor cells (**Figure 4A**). CCK8 assay showed that knockdown of METTL1 significantly inhibited the cell viability of A875 and A375 cells (**Figure 4B**), and significantly inhibited the migration (**Figures 4C, D**) and invasive ability of A875 and A375 cells (**Figures 4E, F**), and also had slight alterations on cell cycle (**Supplementary Figure S3**).

### METTL1 functions as a marker of immunotherapy response in SKCM

**Figure 5:**
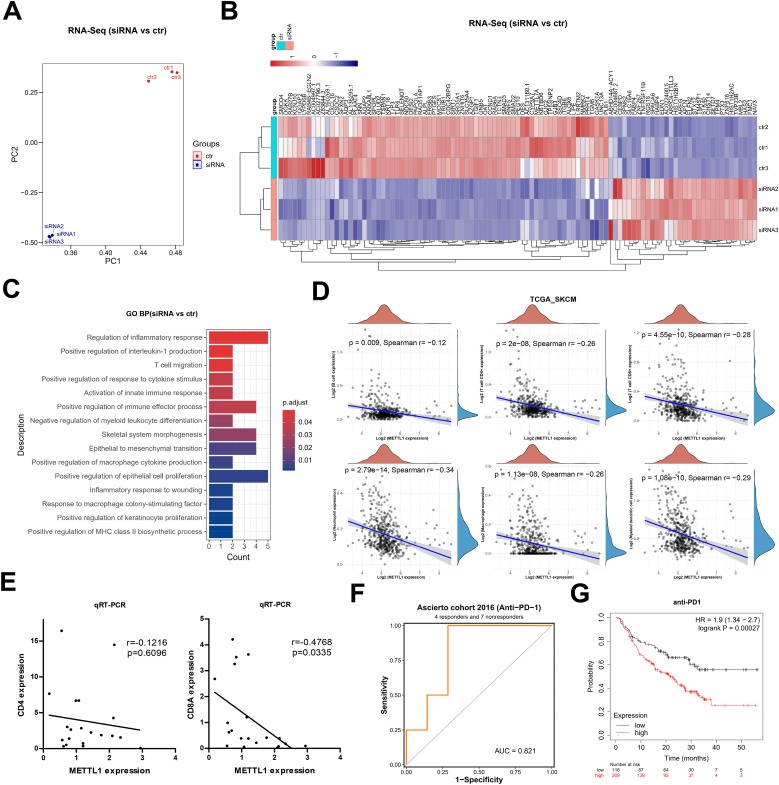
METTL1 as a marker of immunotherapy response in SKCM. **(A)** Principal component analysis of RNA-seq after silencing METTL1. **(B)** Heatmap showed the 103 differentially expressed genes, including 32 up-regulated genes and 71 down-regulated genes. **(C)** GO enriched analysis for these differentially expressed genes. **(D)** The association of METTL1 with B-cell, CD4+ T-cell, and CD8+ T-cell infiltration was analyzed based on the TCGA SKCM cohort data. **(E)** qRT-PCR experiments was performed using SKCM samples to detect the expression of CD4 and CD8A, the relationship of the expression of CD4 and CD8A, and METTL1 was analyzed. **(F)** the BEST data included a cohort analysis on immune checkpoint therapy was used to analyze the association of METTL1 expression and response to anti-PD1 treatment. **(G)** the Kaplan-Meier Plotter online analysis tool was used to analyze the survival of SKCM patients treated with anti-PD1 with high or low levels of METTL1.

Further, we carried out RNA-seq analysis after silencing METTL1. Principal component analysis showed that samples after silencing METTL1 and samples without silencing METTL1 could be significantly distinguished and were closer to each other in the same group (**Figure 5A**). 103 genes were considerably differentially expressed according to differential analysis, of which 32 had significant up-regulation and 71 had significant down-regulation (**Figure 5B**). These differentially expressed genes were mainly enriched in several immune-related signaling pathways, such as T cell migration, epithelial-mesenchymal transition, and inflammatory response (**Figure 5C**). We hypothesized that METTL1 might be related to immune cell infiltration and the response to immunotherapy since T cell migration and the inflammatory response are crucial for anti-tumor immunity. Then, we analyzed the association of METTL1 with B-cell, CD4+ T-cell, and CD8+ T-cell infiltration based on the TCGA SKCM cohort data. Interestingly, we found that the higher the METTL1 expression, the lower the degree of CD4+ T cell and CD8+ T cell infiltration in SKCM patients (**Figure 5D**). To confirm this phenomenon, we performed qRT-PCR experiments using SKCM samples. We found that the expression of METTL1 was not significantly correlated with the expression of CD4, while it was significantly negatively correlated with the expression of CD8A, a marker for CD8^+^ T cells (**Figure 5E**). This evidence suggested that METTL1 might reduce the chance of cytotoxic T cells killing the tumor by inhibiting the infiltration of CD8^+^ T into the tumor, thereby promoting tumor progression.

Current immune checkpoint therapies for antitumor immunotherapy are precisely those targeting CD8^+^ T cells, and thus we hypothesized that the expression level of METTL1 might be correlated with patients’ response to immune checkpoint therapy. Using the BEST data included a cohort analysis on immune checkpoint therapy, we found that METTL1 expression serves as a reliable predictor for response to anti-PD1 treatment (**Figure 5F**). Furthermore, utilizing the Kaplan-Meier Plotter online analysis tool to analyze data from a cohort of SKCM patients treated with anti-PD1 revealed that those with high levels of METTL1 had significantly worse prognosis (**Figure 5G**). These findings suggest that METTL1 may be a pan-cancer immunotherapeutic response marker, and marker studies targeting METTL1 with expanded sample size and tumor type are important for monitoring the clinical efficacy of anti-PD1 immunotherapy.

### High expression of METTL1 may be driven by copy number amplification

**Figure 6:**
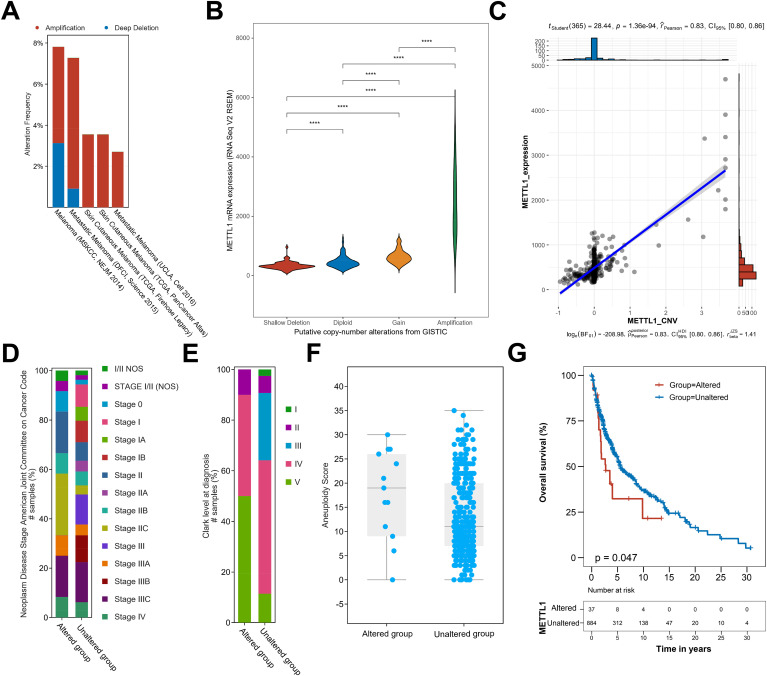
Copy number amplification induces METTL1 expression. **(A)** The genome sequencing data of SKCM cohorts included in the cBioportal database was used to analyze the copy number amplification of METTL1. **(B)** The association of genomic copy number amplification and the mRNA level of METTL1. **(C)** The association of the copy number value of METTL1 and the mRNA level of METTL1. **(D, E)** The percentage of samples with various stage in altered or unaltered METTL1 copy number group. **(F)** The aneuploidy score in altered or unaltered METTL1 copy number group. **(G)** The overall survival in altered or unaltered METTL1 copy numbers group. ****P < 0.00001.

Copy number variation directly affects the expression level of the genes it covers, and there are a large number of copy number variation events in SKCM. Therefore, we conjectured that the high expression of METTL1 is probably driven by an increase in its genomic copy number amplification. Based on this, we analyzed the genome sequencing data of several SKCM cohorts included in the cBioportal database. We found that METTL1 was amplified to varying degrees in all five cohorts (**Figure 6A**), and the mRNA level of METTL1 increased with the increase in the degree of METTL1 genomic copy number amplification (**Figure 6B**), and the copy number value of METTL1 was also significantly and positively correlated with the mRNA level of METTL1 (**Figure 6C**), which strongly suggests that the copy number amplification of METTL1 is what drives its expression. In addition, more the copy number value of METTL1 was altered in high SKCM stage (**Figures 6D, E**). Previous work has identified elevated tumor aneuploidy as a marker of low overall survival and can be used as a biomarker for clinical outcomes of immunotherapy (12), and we found that the copy number value of METTL1 altered samples had higher aneuploidy score (**Figure 6F**). In addition, We found that patients with altered METTL1 copy numbers also had worse overall survival (**Figure 6G**), suggesting that METTL1 be used as a prognostic indication for SKCM.

### MYC was a potential transcription factor of METTL1 in SKCM

**Figure 7:**
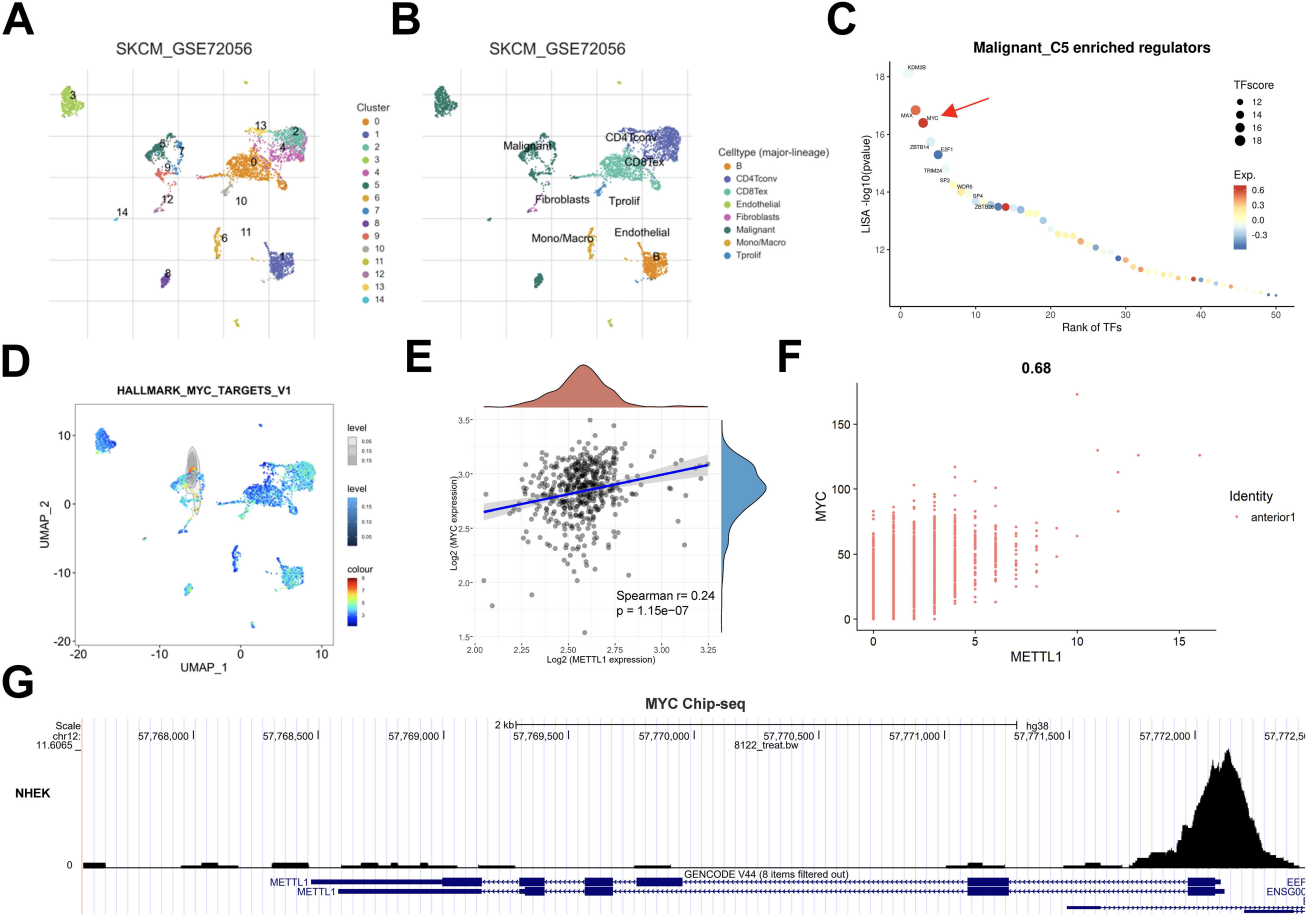
MYC was a potential transcription factor of METTL1 in SKCM. **(A, B)** The SKCM single-cell sequencing data of GSE72056 from the TISCH online database was used to analyze the cell type annotation. **(B)** Transcriptional regulator analysis was performed on the subpopulations that specifically highly expressed METTL1. **(D)** MYC transcriptional regulators are also expressed at higher levels in this cell subpopulation C5. **(E, F)** The association of the expression levels of MYC and METTL1. **(G)** Analysis of Chip-seq data from human epidermal keratinocytes also showed that MYC peak was significantly enriched near the METTL1 promoter.

Disturbance in transcriptional regulatory networks is a common feature of multiple tumors, and activation of oncogenic transcription factors leads to a wide range of downstream effects. We predicted that METTL1 should also be activated by some specific transcription factors. To explore the transcriptional regulatory mechanism of METTL1, we downloaded the single-cell sequencing data of GSE72056, a group of SKCM, from the TISCH online database for analysis. After cell type annotation using this tool (**Figures 7A, B**), transcriptional regulator analysis was performed on the subpopulations that specifically highly expressed METTL1, and we found that MYC transcriptional regulators were significantly enriched (**Figure 7C**), MYC transcriptional regulators are also expressed at higher levels in this cell subpopulation (**Figure 7D**, **Supplementary Figure S4**). Therefore, we hypothesized that MYC may be the most important transcription factor for METTL1. Further analysis of the TCGA SKCM cohort data and SKCM spatial transcriptome data revealed that the expression levels of MYC and METTL1 were significantly positively correlated (**Figures 7E, F**), consistent with the regulatory relationship between transcription factors and target genes. Analysis of Chip-seq data from human epidermal keratinocytes also showed that MYC peak was significantly enriched near the METTL1 promoter (**Figure 7G**). These data suggest that MYC may be a potential transcription factor of METTL1.

## Discussion

RNA modification is a type of epigenetic modification that can play an important role in regulating biological processes and tumor pathology by enhancing the stability and expression of oncogenic transcripts (13). The RNA modifications include N6-methyladenosine (m6A) modification, N1-methyladenosine (m1A) modification, 5-methylcytosine (m5C) modification, 7-methylguanosine (m7G) modification, etc. (14) RNA modification can be mediated by methyltransferases, such as METTL1, METTL3, METTL16, and demethylating enzymes, such as FTO, ALKBH5. RNA modifications can be catalyzed, erased and recognized by methyltransferases such as METTL1, METTL3, METTL16, and accurately regulate the process of methylation, which plays an important role in the proliferation, metastasis, invasion, apoptosis, autophagy, and drug-resistance of tumor cells (15). m7G modification has been found firstly in the initial site of mRNA, and then in rRNAs and tRNAs (16). The m7G modification is a common 5′-modification of mRNAs and an internal modification of various non-coding RNAs. m7G modification is mediated in tRNAs by the METTL1 and WD repeat domain 4 (WDR4) complex, which is significantly involved in various tumorigenesis (6).

METTL1 is often aberrantly expressed and catalyzes m7G modification in tRNAs or miRNAs, which ultimately affects the expression of target genes and regulates tumor-related biological functions (17). It has been shown that the oncogenic function of METTLl can promote tumor cell proliferation and migration by inhibiting the PTEN-related signaling pathway, and that inhibition of METTL1/WDR4 activity reduces m7G tRNA modification and slows down the progression of hepatocellular carcinoma (18). In lung adenocarcinoma and squamous carcinoma, the expression levels of METTLl and WDR4 were significantly elevated compared with those in normal lung tissues, and were closely associated with poor prognosis of lung cancer patients (19). In this study, data mining of public databases revealed that METTL1 was abnormally highly expressed in SKCM and was a poor prognostic factor. The high expression of METTL1 may be associated with abnormal copy number amplification and positive MYC regulation.

We further analyzed and found that METTL1 was co-expressed with stem cell markers in SKCM with positive correlation; knockdown of METTL1 significantly inhibited the clone formation ability of SKCM, suggesting that METTL1 has the function of regulating SKCM stem cells. METTL1 has been shown to have an important role in embryonic stem cell self-differentiation and neural differentiation (20, 21). METTL1 silencing leads to alteration of the entire m7G profile in human induced pluripotent stem cells (hiPSCs) and reduces the translation efficiency of stem cell marker genes. hiPSCs with METTL1-knockdown exhibited reduced totipotency and a slower cell cycle (22). In addition, METTL1 silencing accelerated the differentiation of hiPSC to the embryoid body and promoted the expression of mesoderm-related genes. Similarly, METTL1 knockdown enhanced teratoma formation and mesodermal differentiation *in vivo* by promoting cell proliferation and angiogenesis in nude mice (22). These results suggest that METTL1 plays an important role in the malignant phenotype of SKCM stem cell-like tumor cells.

By mining the RNA-sequencing data after interfering with METTL1 as well as public data, we found that METTL1 functions as a marker of immunotherapy response in SKCM. Polymorphonuclear myeloid-derived suppressor cells (PMN-MDSCs) were enriched in advanced intrahepatic cholangiocarcinoma and significantly correlated with METTL1 (23). Zeng et al. found that liver-specific overexpression or knockdown of METTL1 significantly affected the accumulation of PMN-MDSCs and subsequently affected CD8 + T cell infiltration (24). It was found that the lower infiltrating levels of CD8+ T cells was found in clinical adrenocortical carcinoma samples with high METTL1 expression (25). In this study, we found that the expression of METTL1 was significantly negatively correlated with the expression of CD8A, a marker for CD8+ T cells. These findings suggested that METTL1 might reduce the chance of cytotoxic T cells killing the tumor by inhibiting the infiltration of CD8+T into the tumor, thereby promoting tumor progression. In addition, METTL1 expression serves as a reliable predictor for response to anti-PD1 treatment. Gao et al. found that tumor cell lines with higher METTL1 expression were more sensitive to drugs targeting chromatin histone methylation, ERK-MAPK and WNT signaling pathways (26). *CXCL8* in human and *Cxcl5* in mouse are key translational targets of METTL1 that facilitate its function in promoting PMN-MDSC accumulation in tumor immune microenvironment of intrahepatic cholangiocarcinoma. Co-blockade of METTL1 and its downstream chemokine pathway enhances the anti-PD-1 efficacy in ICC preclinical mouse models (23). METTL1 mediates m7G methylation of PKM mRNA and enhances the expression of its encoded PKM2, while increased PKM2 dimer expression and nuclear translocation activated CD155 expression and induced colorectal cancer immune evasion (27). RNA methylation contributes to revealing the underlying mechanisms of many aspects of tumors, involving initiation, development, invasion, infiltration, and so on. The excessive m^7^G modification of certain genes leads to the acceleration of tumor development. METTL1-mediated m^7^G acts on different RNA targets, affecting the processes of tumorigenesis and immune response. These findings suggest that METTL1 may be a pan-cancer immunotherapeutic response marker, and marker studies targeting METTL1 with expanded sample size and tumor type are important for monitoring the clinical efficacy of anti-PD1 immunotherapy.

Our study relies heavily on data mining of publicly available databases. Although we preliminarily confirmed the oncogenic function of METTL1 in SKCM through cell biology experiments, the results inevitably exhibit some bias due to the small sample size and heterogeneity among samples. We hypothesized through public databases that METTL1 is positively regulated by MYC, but this hypothesis has not been supported by experimental data and requires further clarification. Additionally, we found that SKCM patients with high METTL1 expression had a worse prognosis after anti-PD1 immunotherapy. In light of the existing report that co-blockade of METTL1 and its downstream chemokine pathway enhances the anti-PD-1 efficacy in ICC preclinical mouse models (23). Thus, our inference is highly likely to be reliable, but still requires further confirmation through *in vivo* experiments. The specific mechanisms downstream of METTL1 also need to be validated through single-cell multi-omics analysis of human tissue samples and preclinical animal models.

## In conclusion

The expression of METTL1 was markedly up-regulated in SKCM, and high expression of METTL1 was linked to poor prognosis for SKCM patients, which could serve as an independent prognostic indicator of METTL1. In addition, METTL1 promotes the malignant phenotypes of proliferation, migration, and invasion in SKCM, and may also impede the infiltration of CD8+ T cells into the interior of the tumor by enhancing the communication between tumor cells and fibroblasts and thus forming a physical barrier. Most interestingly, SKCM patients with high METTL1 expression had a worse prognosis after anti-PD1 immunotherapy; hence, it may be a potential biomarker for anti-PD1 immunotherapy in SKCM patients.

## Data Availability

Publicly available datasets were analyzed in this study. This data can be found here: TCGA: TCGA-SKCM; GEO: GSE46517, GSE98394, GSE190113, GSE72056, GSE174401 and GSE115978. cBioPortal: Metastatic Melanoma (UCLA, Cell 2016), Skin Cutaneous Melanoma (TCGA, Firehose Legacy), Skin Cutaneous Melanoma (TCGA, PanCancer Atlas), Melanoma (MSK, NEJM 2014), Metastatic Melanoma (DFCI, Science 2015).
