# Supplementary material for: Upregulated m7G methyltransferase METTL1 is a potential biomarker and tumor promoter in skin cutaneous melanoma
Source: Front Immunol. 2025 May 15;16:1575219. doi: 10.3389/fimmu.2025.1575219 (PMC12119543; doi:10.3389/fimmu.2025.1575219)
Supplement: Supplementary file 1 [file DataSheet1.pdf]

# Upregulated m7G methyltransferase METTL1 is a potential biomarker and tumor promoter in skin cutaneous melanoma

LuLing Xia[1], Ping Yin[2*]

Department of Respiratory, The 3rd Xiangya Hospital, Central South University, Hunan, 410013, China.

Department of Blood Transfusion, The 3rd Xiangya Hospital, Central South University, Hunan, 410013, China.

*Corresponding author, Dr. Ping Yin,

Department of Blood Transfusion, The 3rd Xiangya Hospital, Central South University, Address: Tongzipo Road 138, Changsha, Hunan, 410013, China

E-mail: 51440456@qq.com

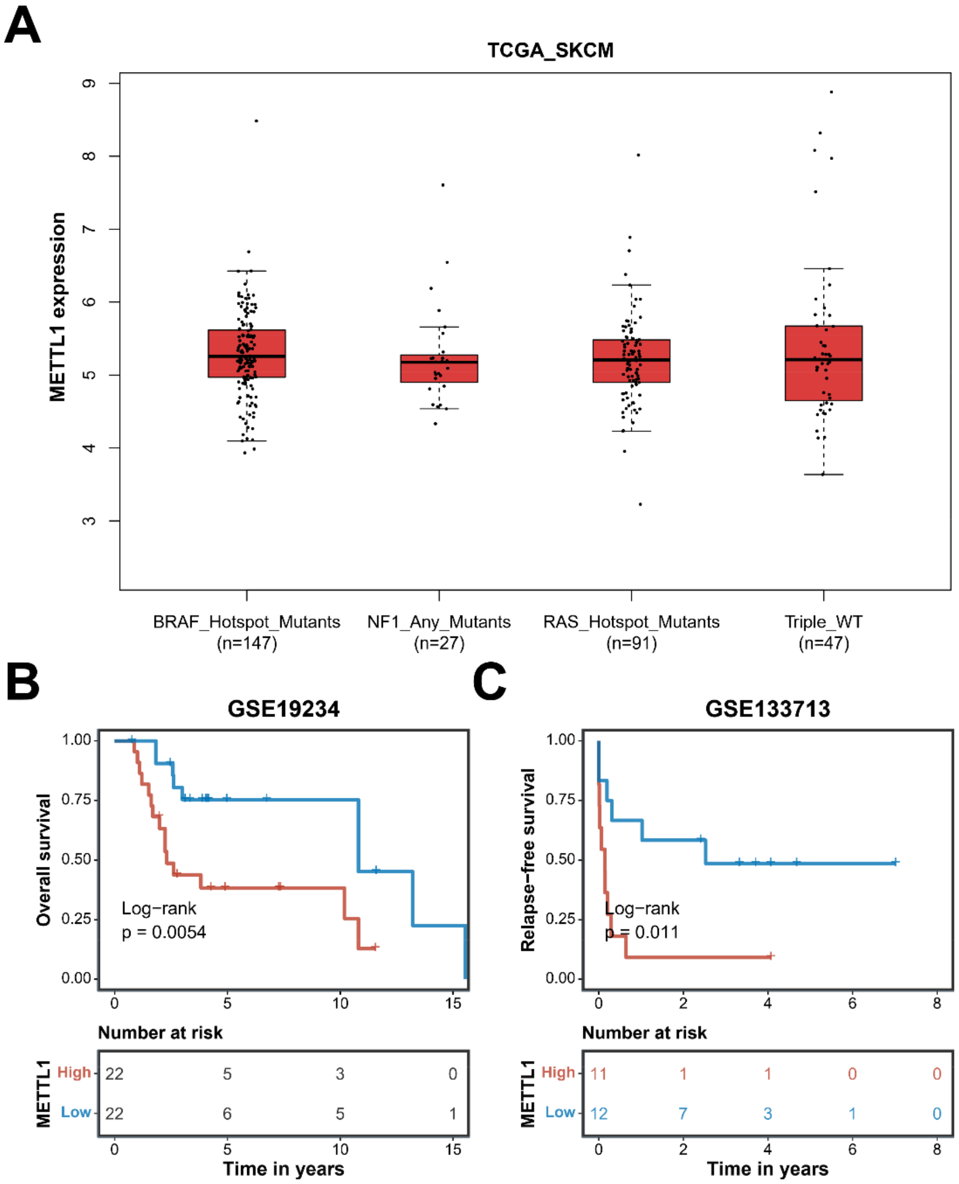


Figure S1 (A) METTL1 expression in four types of SKCM samples, including BRAF mutated, NF1 mutated, RAS mutated, and wild-type without any of the three mutations. (B) Survival analysis in patients with high METTL1 expression in SKCM cohorts, GSE19234. (C) patients with high METTL1 expression in the GSE133713 dataset had lower recurrence-free survival.

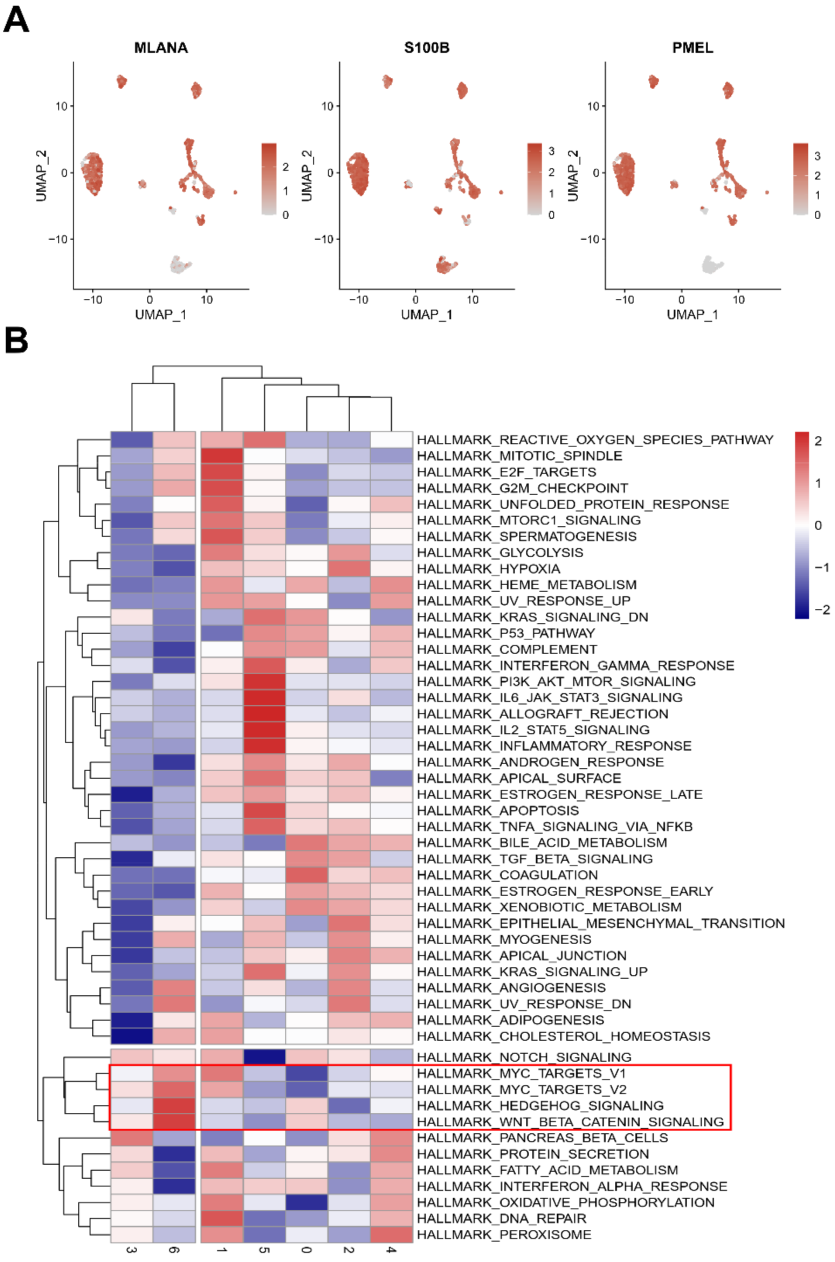


Figure S2 (A) Demonstration of tumor cell marker gene expression from GSE72056 dataset. (B) Heatmap of HALLMARK gene set enrichment analysis for 7 tumor cell clusters in GSE72056.

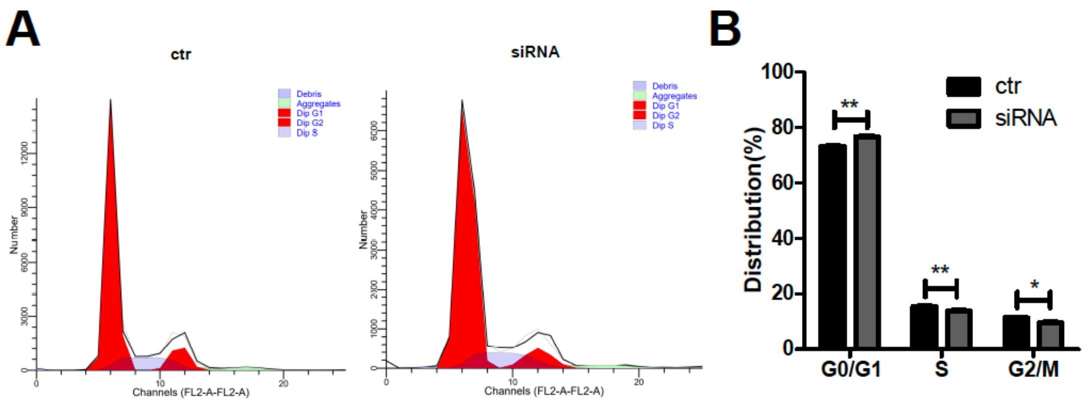

Figure S3 (A) The cell cycle analysis in A875 cells after siRNA transfection, and quantification (B).

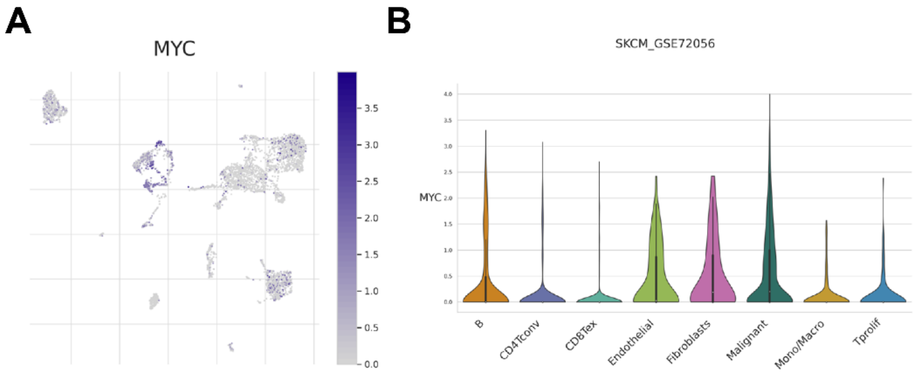

Figure S4 (A-B) The SKCM single-cell sequencing data of GSE72056 from the TISCH online database was used to analyze the expression of MYC in different cells.
